# Survey on reporting of epithelial cells in urine sediment as part of external quality assessment programs in Brazilian laboratories

**DOI:** 10.11613/BM.2021.020711

**Published:** 2021-06-15

**Authors:** José A. T. Poloni, Adriana de Oliveira Vieira, Caroline R. M. dos Santos, Ana-Maria Simundic, Liane N. Rotta

**Affiliations:** 1 Health School, Universidade do Vale do Rio dos Sinos, São Leopoldo, Brazil; 2 Controllab, Rio de Janeiro, Brazil; 3 Department of Medical Laboratory Diagnostics, University Hospital “Sveti Duh”, Zagreb, Croatia; 4 Faculty of Pharmacy and Biochemistry, University of Zagreb, Zagreb, Croatia; 5 Department of Diagnostic Methods and Post Graduation Program in Health Sciences, Universidade Federal de Ciências da Saúde de Porto Alegre, Porto Alegre, Brazil

**Keywords:** urinalysis, External Quality Assessment Program, epithelial cells, survey

## Abstract

**Introduction:**

Epithelial cells (ECs) are structures regularly observed during urine microscopy analysis. The correct identification of EC subtypes can be useful since renal tubular epithelial cells (RTECs) are clinically relevant. We investigate the urinary ECs report and the judgement of its clinical importance by Brazilian laboratories.

**Materials and methods:**

A survey with four questions was made available to participants of the Urinalysis External Quality Assessment Program (EQAP) from Controllab. Laboratories composed 3 groups: (1) differentiating ECs subtypes: “squamous”, “transitional” and “RTECs”; (2) differentiating ECs subtypes: “squamous” or “non-squamous” cells; (3) without ECs subtype identification. Participants did not necessarily answer to all questions and the answers were evaluated both within the same laboratory’s category and within different categories of laboratories.

**Results:**

A total of 1336 (94%) laboratories answered the survey; Group 1, 119/140 (85%) reported that ECs differentiation is important to the physician and 62% want to be evaluated by EQAP, while in Group 3, 455/1110 (41%) reported it is useful to them, however only 25% want be evaluated by EQAP. Group 2 laboratories 37/51 (73%) reported that the information is important, but only 13/52 (25%) are interested in an EQAP with differentiation of the 3 ECs subtypes.

**Conclusion:**

Most of the laboratories do not differentiate ECs in the three subtypes, despite the clinical importance of RTECs. Education of laboratory staff about the clinical significance of urinary particles should be considered a key priority.

## Introduction

Urine sediment analysis requires proper training, equipment, and methodology. The use of automated systems is becoming more easily available to laboratories contributing to an improved workflow. However, manual microscopy is still the gold standard method and is required for samples that present pathological profiles, especially to the identification of the different types of urinary casts, crystals, and also to properly identify the types of cells. External quality assessment programs (EQAP) have a central role in laboratories contributing to the improvement of the quality of the information reported on patient’s test results (*1*).

Epithelial cells (ECs) are an important component of the urine sediment report and can be normal or pathological. The knowledge of the different subtypes of ECs (squamous, transitional, and renal tubular) and their clinical significance are mandatory to professionals dealing with urine microscopy analysis; for example, squamous epithelial cells are a normal find, however, renal tubular epithelial cells (RTEC) are a sign of tubular injury, and can be originated in different parts of the nephron, depending on the site of the injury (*2*). Another important point is that ECs quantities furnish different information about the clinical picture of the patient. Large numbers of squamous ECs are a sign of an improperly collected sample, but large numbers of RTECs are a sign of acute tubular injury, sometimes linked to acute kidney injury. Then, urinalysis (dipstick and microscopy) can be very useful to identify the site of injury, making it possible to know if there is a glomerular, tubular, or interstitial involvement (*2*-*5*).

Most importantly, RTECs are a marker of tubular damage and, coupled with RTEC casts and granular casts, reflect cell death and apoptosis that would be associated with more severe renal tubular injury, and thus worse outcomes. Renal tubular epithelial cells are considered a marker of acute kidney injury associated with acute tubular necrosis (ATN), while deep transitional cells usually indicate severe damage of the uroepithelium which covers the urinary excretory system (*6*, *7*). It was demonstrated the possibility to differentiate prerenal acute kidney injury from ATN using a urine sediment score based on the quantification of RTECs and granular casts (*6*).

On the other hand, the identification of RTECs and the differentiation of RTECs from cells of other parts of the urinary tract is sometimes difficult. Due to these difficulties, some laboratories choose the option to report ECs without making any distinction between the different cell types (*8*, *9*).

Despite the clinical relevance to report the presence of RTECs, the knowledge to properly identify this particular type of cell in routine urinalysis was never addressed in a published work with Brazilian laboratories. Also, it is important to evaluate how laboratories deal with the “epithelial cell” information during their routine work.

To the best of our knowledge, this is the first survey on how clinical laboratories report ECs during routine urinalysis. This brings light to this information that shows clinical interest. This paper aimed to investigate how Brazilian clinical laboratories report the microscopic findings of urine ECs and how they judge their clinical relevance in diagnosing kidney and urinary tract diseases.

## Materials and Methods

### Study design

A survey with four questions was performed in 2018, May, to Brazilian laboratories that participate in the Urinalysis EQAP from Controllab, a partner of Sociedade Brasileira de Patologia Clínica/Medicina Laboratorial. The survey was made available to all participants on Controllab’s online system, by accessing the form to report the results of 2018, May round. The specialists in laboratory medicine were asked to provide information (one answer per laboratory only) about their laboratory/institution and the urinalysis routine, the policy for reporting the ECs in the laboratory report, and their opinion about the clinician knowledge on the different epithelial cells subtypes importance.

Participants were asked to choose only one of the possible answers to each question and the laboratories were stratified into three different categories/Groups, according to the way they report the ECs: (a) laboratory reports the three cell subtypes: “squamous”; “transitional”; “renal tubular epithelial cells”; (b) laboratory reports two cell subtypes: “squamous”; “non-squamous”; (c) laboratory reports all epithelial cells in just one category, as “epithelial cells”. The answers to the questions were evaluated both within the same laboratory’s category and within different categories of laboratories. Not all laboratories answered all the questions.

### Statistical analysis

Data are presented as counts and percentages. All calculations were done in Microsoft Excel 16.0 (Microsoft, Redmond, USA).

## Results

A total of 1336/1412 (94%) Brazilian medical laboratories responded to the questions of the survey, being 1230 Private institutions and 186 Public institutions, and the expressive majority of them do not differentiate the EC subtypes. The questions with the respective answers are presented in Table 1.

**Table 1:** Summary of the data form questionnaire, according to the ECs reporting group

	**Laboratory category, N (%)**		
	**1 -“squamous”; “transitional” or “RTEC”** **N = 140**	**2 - “squamous” or “non-squamous”** **N = 52**	**3 - “epitlhelial cell”** **N = 1144**
**How do you perform the urine sediment analysis?**			
a) Microscopic analysis	112 (80)	37 (71)	975 (87)
b) Automated analysis and microscopic review when the equipment reports flags	15 (11)	12 (23)	96 (9)
c) Automated analysis (count) and microscopic review	8 (6)	2 (4)	35 (3)
d) Other	5 (3)	1 (2)	8 (1)
**In what situation do you inform the different subtypes of epithelial cells?**			
a) To any sample because it is a standard procedure of the laboratory	133 (96)	44 (88)	0*
b) Only when the equipment flags the sample	4 (3)	3 (6)	0*
c) Only when requested by the physician	2 (1)	3 (6)	0*
**In your opinion, to inform the 3 subtypes of epithelial cells is useful to the physician?**			
a) Yes	119 (85)	37 (73)	455 (41)
b) No	15 (11)	12 (23)	652 (59)
c) Other: believe physicians are not familiar to use this kind of information	6 (4)	2 (4)	3 (0)
**Is your laboratory interested in evaluation of the performance of the correct identification of the different cells subtypes by an EQAP?**			
a) Yes, evaluating the 3 different subtypes	86 (62)	13 (25)	282 (25)
b) No	51 (36)	19 (37)	782 (68)
c) Yes, but evaluating cells only as squamous or non-squamous	3 (2)	20 (38)	80 (7)
*Laboratory does not inform different subtypes of cells. Not all laboratories answered all the questions. Analysis is presented within the same category of laboratories. ECs – epithelial cells. RTEC – renal epithelial tubular cell. EQAP – external quality assessment program.			

Groups 1, 133/139 (96%), and 2, 44/50 (88%) differentiate the urine EC types as their laboratory routine. Approximately half of laboratories 679/1301 (52%) reported that to inform the three ECs subtypes is not an important information to furnish to the physician. On the other hand, 492/1161 (42%) laboratories from Groups 2 and 3, stated that it is an important information, while 664/1161 (57%) reported otherwise; up to 6/140 (4%) of laboratories, independently the way they report ECs, stated that the physicians are not familiar to use this kind of information. Instead of this, the major part of laboratories that perform ECs differentiation stated that informing the three subtypes of ECs is useful to the physician.

The majority of laboratories 1124/1306 (86%) perform the urine sediment analysis by microscopy, without the aid of any kind of automation.

The interest to evaluate the laboratories’ performance about the ECs identification was also verified. In Group 1, the major part of laboratories stated that the ECs differentiation is important to the physician and also reported that it is important to participate in EQAP that evaluates the three EC subtypes. However, In Group 3, 455/1110 (41%) laboratories reported that EC differentiation is useful to the physician and only 282/1144 (25%) of them are interested in being evaluated by EQAP. From Group 2, 37/51 (73%) stated that the information is important, but only 13/52 (25%) are interested in an EQAP with differentiation of the three ECs subtypes, while 20/52 (38%) are interested in an EQAP that evaluates the cells in the way they routinely identify, *i.e.*, only as “squamous” or “non-squamous”.

## Discussion

This study provides an insight into how clinical Brazilian laboratories report the microscopic findings of urine ECs, as well as what is their view about the clinical relevance of ECs in diagnosing kidney and urinary tract diseases.

The results show evidence of the lack of knowledge of laboratory professionals about the different types of ECs and their clinical significance since the major part of laboratories reported that to inform the three subtypes of ECs is not an important information to furnish to the physician. The consequence of the lack of knowledge on these types of cells clinical significance can potentially lead to improper result reports without relevant information, like the presence of RTECs, contributing to delay in the diagnosis of important clinical conditions.

Based on the fact that the RTECs are a well-established marker of tubular injury that can be identified in the urine sediment, the opinions reported by the major part of Brazilian laboratories are contrary to the knowledge available in the literature, because the major part of the laboratories from Group 3 reported that inform the three ECs subtypes is not an important information to the physician (*6*, *7*). On the other hand, the major part of laboratories from Group 1 understands the importance of this information to the physician.

Then, the Brazilian laboratories were asked about their interest in EQAP focusing the EC analysis. Most laboratories don’t differentiate ECs subtypes and reported that it is not useful for the physician and has no interest in being evaluated on the identification of different cell subtypes using an EQAP. Instead of this, the major number of the laboratories from Group 1 are interested in evaluating the performance by EQAP, to the correct identification of the different ECs subtypes.

Urine microscopy professional training is a difficult task. Quality control programs have a central role in the improvement of the knowledge and on the continuing education of laboratory professionals. External Quality Assessment Program on urinary sediment are still rare despite the fact it is mandatory for accreditation programs in Laboratory Medicine (*1*). The literature about the EQAP on urinalysis is very few for test strips or analytes of quantitative clinical chemistry. More recently, studies of EQAP in urine, only dealing with sediment examinations, were published in the United States (US) and some countries of Europe (*1*).

External Quality Assessment Programs are an essential tool to assist with the technology, identify problems, and to point training needs and it is valuable for laboratories, but even more for the benefit of the patients (*10*, *11*). Studies have reported the importance of continuing education and regulatory supervision in contributing to improve the performance in EQAP, and to decrease the number of errors in urine analysis (*12*, *13*). Failing laboratories must analyse the reasons for the failure, report the results, and initiate corrective action. Over the years, there has been a progressive decline in the number of errors, demonstrating that education and regulatory oversight are major contributors to improved EQAP performance and, by extension, patient care (*14*, *15*).

A Northern European experience with 329 participating laboratories in an EQAP of urine particle identifications, based on images, showed the following result to the EC identification: squamous EC 92–98%, and small EC 73–83% (minimum and maximum of expected or accepted reports). To EC identification, abundant squamous ECs were demonstrated together with other smaller transitional ECs in the same image. However, the identification of smaller ECs was more difficult: only 71% of participants reported the expected “transitional EC”, an additional 5% generally identified it as “small EC” at the basic level, and 4% of participants as “atypical EC” that were considered acceptable due to degeneration of the particles (*13*).

Secchiero *et al*. describe the results obtained in the Italian EQAP on urinary sediment with the use of images to supply identification and clinical association of urine sediment particles. For the cells, the highest rate of correct identification was for squamous EC and dysmorphic erythrocytes (~97%) while the lowest were acanthocytes and macrophages (~57%), and intermediate were RTECs (~72%). Epithelial cells such as RTECs and transitional cells, both, superficial and deep, were correctly identified by about 70% of participants. For the transitional ECs, the participants observed whether they derived from the superficial or deep layers of the uroepithelium, a fact that caused several partially correct answers (*1*).

Laboratory professionals need to understand the relevance of the information that can be reported, independent of the test that is being performed. Informing the different EC types can contribute a lot to the clinical management of patients especially when RTECs are observed during routine analysis. However, the “Continuing educational activities” should also bring out the need for clinician orientation by laboratory professionals. The clinical-laboratory interface is becoming appreciated as an under-recognized yet vital component of quality healthcare delivery in all fields. Without improved clinical knowledge, improving laboratory quality can only solve part of diagnostic accuracy (*16*).

This study has some limitations: a) no images of different ECs were sent to evaluate the knowledge of the laboratory professionals on the correct identification of these types of urinary particles; b) it is a survey, so, no sample was performed to evaluate the technical performance of laboratory professionals; c) the work was produced after the observation that the results obtained with the survey were useful to the literature, so, the original survey was not planned to be an article originally – it can explain some down points of the study design.

Continuing educational activities conducted during the rounds of the EQAP with information of urinary particles, especially, in this case, focusing on the different types of ECs and their clinical significance could be a contributing addition to the evolution of knowledge about the particles and consequently leading to an improvement of the quality of the test reports and professionals’ opinions.

In conclusion, most of the laboratories do not differentiate the ECs in the three subtypes, despite the importance of RTECs as a marker of renal tubular injury. Laboratories that already work on a higher level of quality information (the minority) reporting different ECs subtypes want to be evaluated by EQAP, while those that work with a lower level of quality information (not differentiating EC subtypes) stated that this information is not useful to clinicians and don’t want to be evaluated by EQAP. Education of laboratory staff about the clinical significance of urinary particles should be considered a key priority.
